# The functional characteristics of optogenetic gene therapy for vision restoration

**DOI:** 10.1007/s00018-020-03597-6

**Published:** 2020-07-29

**Authors:** Moritz Lindner, Michael J. Gilhooley, Stuart N. Peirson, Steven Hughes, Mark W. Hankins

**Affiliations:** 1 The Nuffield Laboratory of Ophthalmology, Sleep and Circadian Neuroscience Institute, Nuffield Department of Clinical Neurosciences, University of Oxford, Oxford, UK; 2 Oxford Eye Hospital, Oxford University Hospitals NHS Foundation Trust, Oxford, UK; 3 Institute of Physiology and Pathophysiology, Department of Neurophysiology, Philipps University, Marburg, Germany; 4 Department of Neuroophthalmology, Institute of Ophthalmology, London, UK

**Keywords:** Optogenetics, Vision restoration, Red-shifted channelrhodospin, Retinal degeneration, Gene therapy

## Abstract

Optogenetic strategies to restore vision in patients blind from end-stage retinal degenerations aim to render remaining retinal neurons light-sensitive. We present an innovative combination of multi-electrode array recordings together with a complex pattern-generating light source as a toolset to determine the extent to which neural retinal responses to complex light stimuli can be restored following viral delivery of red-shifted channelrhodopsin in the retinally degenerated mouse. Our data indicate that retinal output level spatiotemporal response characteristics achieved by optogenetic gene therapy closely parallel those observed for normal mice but equally reveal important limitations, some of which could be mitigated using bipolar-cell targeted gene-delivery approaches. As clinical trials are commencing, these data provide important new information on the capacity and limitations of channelrhodopsin-based gene therapies. The toolset we established enables comparing optogenetic constructs and stem-cell-based techniques, thereby providing an efficient and sensitive starting point to identify future approaches for vision restoration.

**Electronic supplementary material:**

The online version of this article (10.1007/s00018-020-03597-6) contains supplementary material, which is available to authorized users.

## Introduction

Degenerative retinal disorders are among the leading causes of blindness in industrial countries [1]. These include the rather common multi-factorial age-related macular degeneration and hereditary diseases of diverse genetic backgrounds like retinitis pigmentosa (RP). Together these diseases represent a major societal and economic burden. With the advent of gene therapy, therapeutic opportunities are now emerging for affected individuals and have even received regulatory approval in some jurisdictions for single conditions [2]. Most current approaches aim to correct single disease-causing genetic defects. These are usually primarily intended to arrest visual loss, however, some degree of functional improvement, presumably by re-activating degenerating photoreceptors, has been reported [3, 4]. A novel strategy involves the expression of transgenes encoding photosensitive proteins (translational optogenetics) in surviving retinal cells such as ganglion cells [5–7], bipolar cells [8–12] or outer segments degenerate photoreceptors [13], to make them directly light sensitive [5–21]. Such an optogenetic gene therapy is particularly attractive as it does not depend on the integrity of the retinal pigment epithelium photoreceptor complex. Thereby it offers the potential to restore vision even in late-stage retinal degenerations, while other approaches aim only to arrest further visual loss [22]. Moreover, it can function independently of the underlying disease cause and could thereby represent an efficient way to offer a mutation-independent therapeutic option even to individuals affected by orphan retinal degenerative disorders.

To date, several groups have successfully shown that delivery of different optogenetic constructs into remaining retinal neurons can restore light responsiveness of retina degenerate rodents [5–21] and these promising results have fostered the initiation of early stage clinical trials (NCT02556736, NCT03326336 at clinicaltrials.gov). Despite these pivotal advances, the amount of information available regarding the extent to which complex image forming vision can be restored at the retinal level, and also what are the likely functional limits remains scarce.

An accurate understanding of both, however, is required to enable efficient design of functional endpoints for clinical trials and allow for direct comparison of the growing number of available candidate optogenetic constructs (ranging from microbial opsins to mammalian opsins and even synthetic opsin-based constructs) before they enter clinical trials (e.g., 9,5,7). Moreover, also optimization of biomimetic goggles that adjust the light stimuli delivered to the retinae of treated individuals (e.g., GD030-MD as employed in NCT03326336) will be facilitated by a better understanding of how optogenetic tools restore complex image forming vison.

In the present work, we established and utilized a series of retina-level functional tests to quantify to what extent responses to complex light stimuli can be restored following intravitreal viral delivery of red-shifted channelrhodopsin (ReaChR) [23, 16] in the *rd1* mouse, an extensively characterized rodent model of human RP [17]. We used viral gene delivery strategies to evoke either retinal ganglion-cell or retinal bipolar cell-dominated expression of ReaChR. Thereby, we were able to restore a variety of light responses closely paralleling those that can be observed in the retina of healthy mice. While highlighting the great potential of optogenetics for vision restoration, the data presented here equally identify functional limitations. Importantly, our approach represents a sensitive toolset to interrogate retinal circuity, compare future candidate optogenetic constructs and treatment time points for vision restoration, and, potentially also evaluate alternative vision-restoring approaches like stem-cell therapy or prosthetic devices at the retinal output level. It can be employed as the first step to efficiently identify promising candidate optogenetic constructs and move them forward towards behavioral analysis and towards Phase I/II clinical trials and reduce unnecessary animal experiments.

## Results

**Fig. 1:**
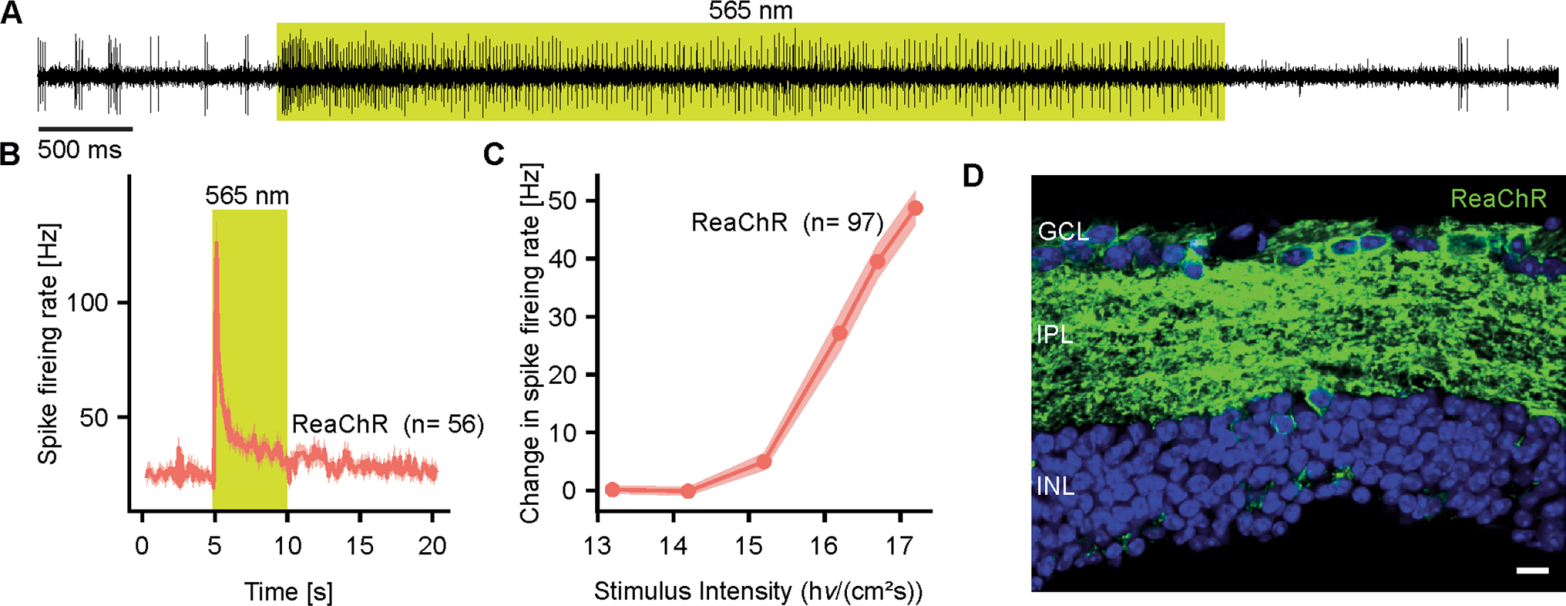
Expression of red-shifted channelrhodopsin (ReaChR) induces fast light responses in degenerate retinae. **a**, **b** Change in spike firing rate in response to light observed in retinae from *rd1* mice after intravitreal delivery of AAV-ReaChR-mCitrine. **a** Representative recordings from a single neuron. **b** Summary time series from 56 individual neurons. Trace represents mean ± standard error of the mean. The lime bar indicates the episode where the retinae were exposed to a full brightness (1.96 × 10^17^ photons × cm^− 2^ s^−1^) 565 nm light stimulus (bar color corresponding to stimulus wavelength). **c** Intensity-response curve as obtained from retinae from treated mice. Half-maximal responses (EC50) were observed at 2.53 (0.64–5.23) × 10^16^ photons·cm^−2^ s^−1^, according to Hill-fits of the individual traces. **d** Representative confocal cross-sectional scan obtained from a retina from a treated *rd1* mouse, immunostained for AAV-ReaChR-mCitrine (anti-YFP, green). Blue: DAPI. *GCL* Ganglion cell layer, *IPL* Inner plexiform layer, *INL* Inner nuclear layer. Scale bar 10 µm. Raw data available as Digital Supplementary Material

To model the extensive photoreceptor loss seen in end-stage RP, the *rd1* mouse was employed. These mice have a non-sense mutation in the *Pde6b* gene, which leads to rapid degeneration of rod photoreceptors followed by cone loss [24]. *rd1* mice were injected with AAV-ReaChR-mCitrine at the age of 6 weeks. Tissue was collected at a median age of 32 (interquartile-range: 27.5–33) weeks and widespread expression of ReaChR-mCitrine was evidenced by anti-YFP immunostaining. Transduced cells were predominantly located in the ganglion cell layer (GCL), but to lesser extent also in the inner nuclear layer (INL). Retinal sections further confirmed the outer retina was fully degenerate (Fig. 1d). Counterstaining against cone-arrestin assures the virtual complete absence of cone photoreceptors (Suppl. Figure 1a, b).

Multi-electrode array (MEA) recordings from retinal explants of mice injected with AAV-ReaChR-mCitrine were performed at the same time points as immunohistochemistry. With the ganglion cell layer exposed to the electrodes, rapid changes in spike-firing rate were observed in response to a 5 s full brightness 565 nm light stimulus in treated mice (Fig. 1a, b). In a lower percentage of electrodes (19% vs. 43%, *p* = 0.01, Chi square test), untreated *rd1* mice also exhibited light responses, resembling photoresponses from intrinsically photosensitive retinal ganglion cells (ipRGCs). As expected, these were significantly lower in frequency [110.00 (85.00–120.00)/s vs. 135.00 (90.00–170.00)/s, *p* = 0.02], slower (Time to peak frequency, median (inter-quartile-range): 5.80 (3.35–6.45) s vs. 0.10 (0.00–0.10)/s, *p* < 0.001] and notably more sustained, with ipRGC responses exceeding the duration of the recordings (Suppl. Figure 2). To further confirm that the observed light responses in treated mice were not governed by intrinsic ipRGC responses, we repeated the above experiments in a cohort of retina degenerate *rd1* mice that additionally lacked *Opn4* expression (*Pde6b*^*rd1/rd1*^*.Opn4*^*−/−*^), which is required for ipRGC light responses [25]. Light responses observed in AAV-ReaChR-mCitrine injected *Pde6b*^*rd1/rd1*^*.Opn4*^*−/−*^ mice were essentially identical to those observed in mice that expressed native melanopsin in terms of peak firing rate [125.00 (107.50–152.5)/s, *p* = 0.11] and onset kinetics [0.10 (0.10–0.30] s, *p* = 0.76, Suppl. Figure 2]. Data from *Opn4*^−/−^ and *Opn4*^+/+^ mice were thus pooled for further analyses.

Irradiance response curves (IRCs) generated for responsive electrodes in retinae following vector delivery revealed dose-dependency of the induced photoresponses with a half-maximal response (EC50) at 2.53 (0.64–5.23) × 10^16^ photons·cm^−2^ s^−1^ (Fig. 1b), consistent with previously published observations in retinal neurons [16]. Notably, this irradiance-response relationship did not only reflect decreased response amplitudes at lower light levels in individual neurons. Also, the portion of neurons responding at all (i.e., showing a change in spike firing rate of ≥ 10 Hz) notably changed: While at 1.96 × 10^17^ photons·cm^−2^ s^−1^ and 5.01 × 10^16^ photons·cm^−2^ s^−1^ for virtually all neurons included into the analysis did actually respond [100 (91.67–100)% and 100 (83.59–100)%, respectively], this portion notably dropped at lower light intensities [64.62 (45.83–93.75) % for 1.96 × 10^16^ photons·cm^−2^ s^−1^].

**Fig. 2:**
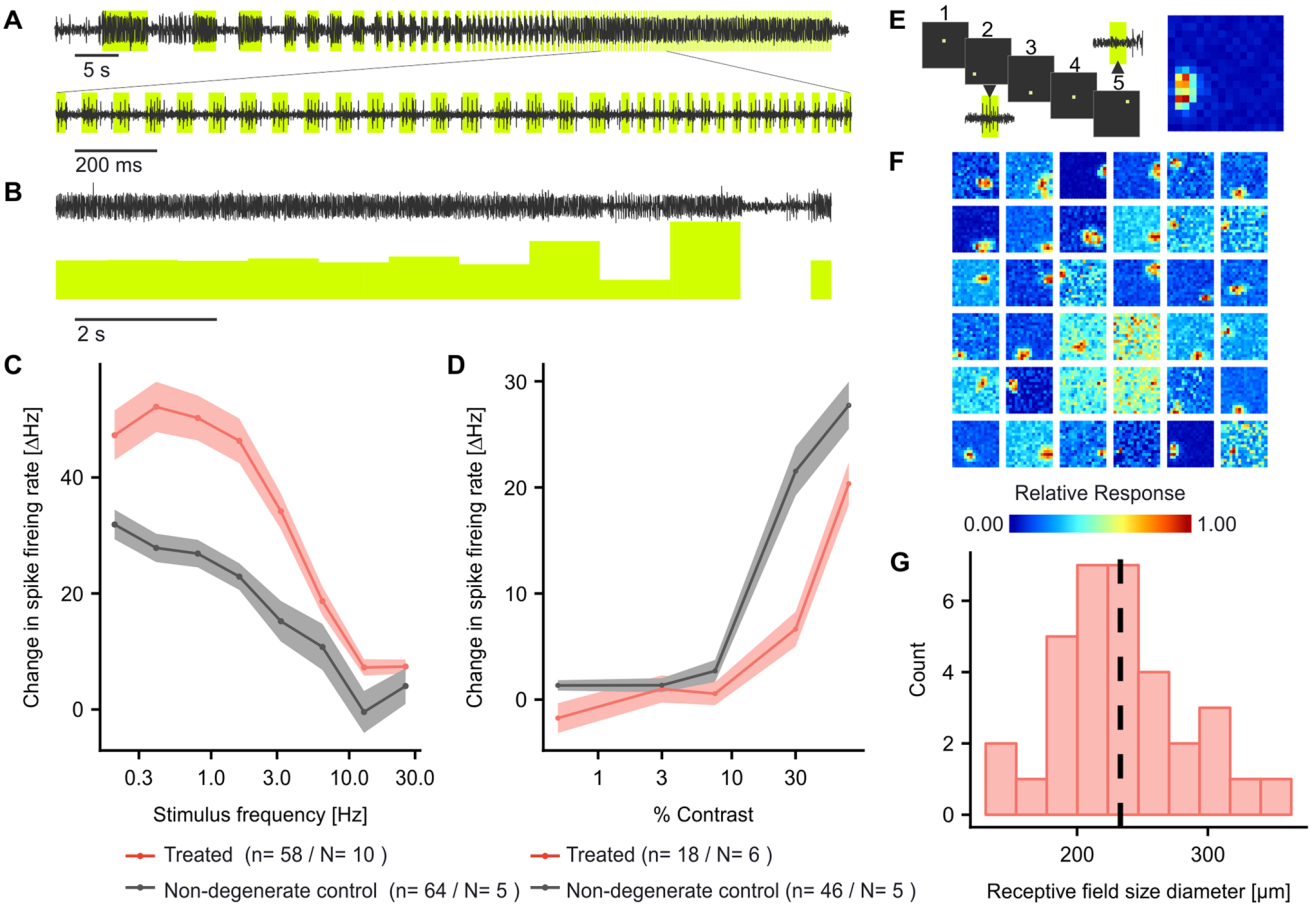
Spatiotemporal characteristics of ReaChR mediated light responses. **a**, **c** Change in spike firing rate in response to a 1.96 × 10^17^ photons × cm^−2^ s^−1^ 568 nm flicker stimulus of increasing frequency as observed in AAV-ReaChR-mCitrine treated degenerate retinae. **a** shows a representative recording from a single neuron, with lime areas indicating times where the light was switched on. **c** summarizes responses recorded from treated degenerate (red) and non-degenerate (grey) retinae. **b** Representative recording obtained from a treated degenerate retina in response to a stepwise contrast modulation on a 9.8 × 10^16^ photons × cm^−2^ s^−1^ 568 nm background. Corresponding summary curves are for treated degenerate (red) and non-degenerate (grey) retinae are shown in D. **e** Receptive field maps (resolution: 2.38 × 10^–3^ mm^2^/pixel) were obtained in response to a sparse binary noise stimulus. The left panel exemplifies five frames of a stimulus sequence. For frame #2 and #5 the neural responses recorded while the stimulus was projected are also shown. In #2, a sharp increase in firing rate can be observed, indicating that the stimulus was projected into the receptive field of that neuron, while this was not the case for the stimulus shown in #5. The right panel shows the resulting receptive field map. **f** Receptive field mappings from each responding neuron as recorded in response to the stimulus paradigm shown in E (intensity: 1.96 × 10^17^ photons× cm^−2^ s^−1^, 568 nm, *n* = 36, *N* = 8). Responses are normalized for each single neuron. **g** Histogram of corresponding receptive field size diameters (derived from area where 95% of the observed spikes occur). Dashed line indicates mean receptive field size. See Suppl. Figure 4 for receptive field maps and receptive field size histograms obtained from non-degenerate untreated mice under identical conditions. Traces in **c** and **d** represent mean ± standard error of the mean

To test to what extent AAV-ReaChR-mCitrine-induced responses could also encode complex visual stimuli, a set of stimulus paradigms was applied. When exposed a full-field bright flicker stimulus of increasing frequency, ganglion cell firing responses could follow the stimuli up to the maximum frequency tested (25.4 Hz, Fig. 2a, c). Note, that while the change in spike firing rate plateaued at frequencies of 12.7 Hz and higher, it was significantly different from zero (*p* < 0.003, One-Sample Wilcoxon test) for the entire range of frequencies tested (0.4–25.4 Hz). For high frequencies, however, this seemed driven by only subsets of cells that were able to encode such high stimulus frequencies (Suppl. Figure 3). We then explored to what extent AAV-ReaChR-mCitrine-treatment was capable of also restoring responses to gradual changes in light intensity. When applying a step-wise contrast modulation protocol, significant changes in spike firing rate from baseline could be observed above 30% contrast (*p* < 0.001 for both, Fig. 2b, d). For comparison, similar recordings were performed in retinae from non-degenerate mice. While in these mice response amplitudes for small contrast changes were weak, even responses to 0.5% contrast were significantly different from zero (*p* = 0.003, One-Sample Wilcoxon test), confirming that the experimental setup would have allowed us to detect responses to such low contrast levels in treated mice, if they had been restored by AAV-ReaChR-mCitrine-treatment.

**Fig. 3:**
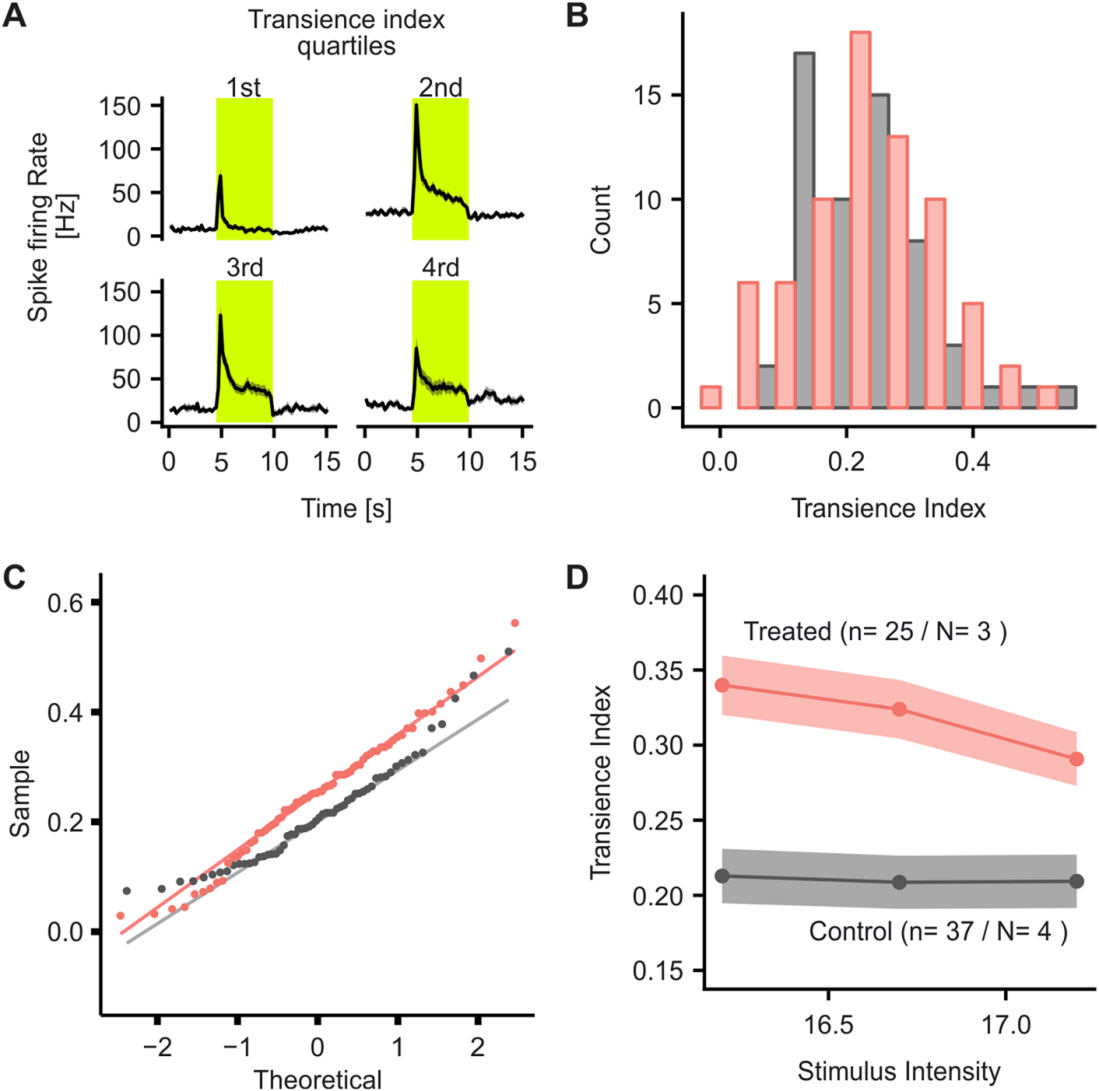
Heterogeneity of response duration observed in AAV-ReaChR-mCitrine treated degenerate retinae. **a** Time traces of spike firing rate in response to a full brightness stimulus (1.96 × 10^17^ photons × cm^−2^ s^−1^, 565 nm), grouped by the transience index of the response. Transience indices range from 0 to one, with one representing a response that is perfectly sustained and equal in amplitude over the entire stimulus duration. **b** Histogram and (**c**) QQ-Plot of the transience indices obtained from individual neurons from AAV-ReaChR-mCitrine treated (red) and wild-type retinae (grey). For AAV-ReaChR-mCitrine treated retinae, histogram and QQ-plot both provide no evidence for a non-normal distribution, while for wild types they do as expected (Shapiro–Wilk: *p* = 0.72 and *p* = 0.006, respectively). **d** Transience of spike firing obtained in response to light stimuli of increasing intensity: In treated degenerate retinae there is a consistent trend that responses become increasingly transient with increasing stimulus intensities (*n* = 25, *N* = 3, *r* = − 0.21, *p* = 0.071) while such a trend cannot be observed in wild-type controls (*n* = 37, *N* = 4, *r* = -0.01, *p* = 0.201)

Next, we addressed the question of whether AAV-ReaChR-mCitrine was capable of restoring spatially precise neural responses in degenerate retina. Using a sparse binary noise protocol (Fig. 2e), defined receptive fields could be mapped from 33 out of 36 responding neurons from treated *rd1* mice (91%, Fig. 2f). Mean receptive field diameter was 222.40 (193.10–252.40) μm (Fig. 2g). For comparison, measuring receptive field diameters in wild-type retinae, using an identical stimulus protocol, the obtained receptive field sizes were in a similar range [306.58 (228.52–331.42) μm, Suppl. Figure 4, *p* = 0,999; one-sided Wilcoxon test for inferiority of the receptive fields obtained in treated mice]. Of note, measures for receptive field diameters in AAV-ReaChR-mCitrine treated retinae were normally distributed, while this was not the case in responses recorded from wild-type retinae (*p* = 0.59 vs. *p* = 0.023), which may reflect the lack of signal processing occurring in the treated retinae. Stimulus intensities employed for wild-type retinae were identical to those used to stimulate the treated degenerate retinae to facilitate direct comparison. A summary of the responses obtained in wild-type retinae to dimmer stimuli is provided in Suppl. Figure  4. In response to sustained stimuli, AAV-ReaChR-mCitrine-induced responses are transient with a rapid reduction in spike firing rate observed in most neurons (Fig. 1a). Either, such transience could reflect the intrinsic membrane properties of the transduced subtypes of RGCs (or RGCs receiving input from another transduced retinal neuron) [16, 6, 8] or they could represent transgene or transgene-delivery related factors, for example, ReaChR inactivation/desensitization or depolarization block induced by excessive activation of ReaChR. To explore which was the case, we grouped neural responses obtained to a sustained stimulus into quartiles based on their transience, revealing a remarkable heterogeneity (Fig. 3a). Transience index ranged from 0.03 – 0.56 [median: 0.25 (0.18–0.32)]. If this heterogeneity was a result of intrinsic membrane properties of distinct subtypes of transduced RGCs transience indices would be expected to be non-normally distributed, according to the discrete relative abundance of the respective RGC subtypes and their defined membrane properties. Indeed, for wild-type controls, the histogram and QQ-plot indicated a non-normal distribution (Shapiro–Wilk *p* = 0.006). By comparison, there was no evidence of any non-normal distribution for AAV-ReaChR-mCitrine treated retinae (Shapiro–Wilk: *p* = 0.58—please note the higher number of individual neurons recorded and analyzed for treated retinae compared to wild types; Fig. 3b, c). Under the same hypothesis, response transience would be expected to be relatively independent of the stimulus intensity, while ReaChR deactivation and a depolarization-block type effect induced by excessive activation of ReaChR should result a response transience that increases with stimulus intensity [23]. We therefore analyzed RGC responses to sustained stimuli of increasing levels of light and observed that in treated retinae spike-firing responses did become more transient with increasing stimulus intensities (*n* = 25, *N* = 3, *r* = − 0.2, *p* = 0.07). By contrast, recordings from wild-type retinae showed constant transience indices over the same range of stimulus intensities (*n* = 37, *N* = 4, *r* = − 0.01, *p* = 0.201, Fig. 3d). Collectively, these results indicate that response transience may be rather due to the transgene or transgene-delivery-related factors rather than physiological membrane properties of the transduced RGC subtypes.

**Fig. 4:**
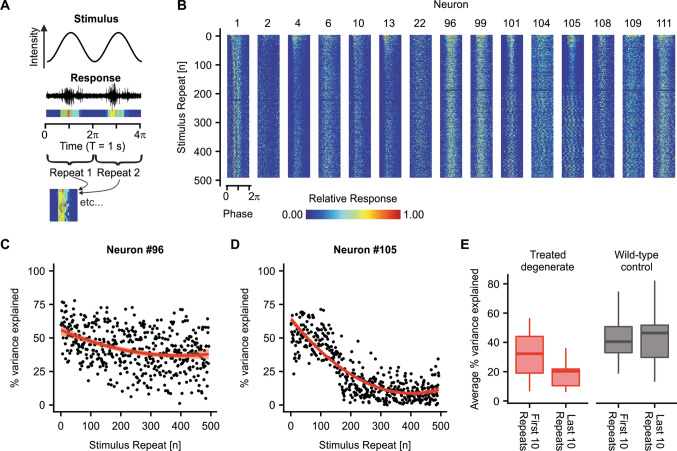
Long-term responsiveness in AAV-ReaChR-mCitrine treated retinae to quasi-realistic light stimuli. **a** Schematic illustrating stimulus shape and processing of the recorded neural responses. A continuous cosine intensity modulated full field stimulus (*T* = 1 s, peak intensity: 1.96 × 10^17^ photons × cm^−2^ s^−1^, 565 nm) was displayed over 500 repeats. Binned spike firing responses were normalized, colour coded for visualization (dark blue: minimum firing rate, red: maximum firing rate) and analysed in a stimulus phase-aligned manner. **b** Raster-plot for individual neurons (recorded from *N* = 3 retinae). Spike firing responses in some neurons rapidly wear off (e.g. #2) or become less defined (e.g., #101, #105) while others appear stable (e.g., #2). **c**, **d** Percentage of variance explained (%VE) over time in the neural responses, obtained by fitting the neural responses of each phase-repeat to a cosine-function as underlying the stimulus. **c** Example of a neuron with relatively stable and **d** unstable responsiveness over time. Red line: LOESS-regression curve. Note that the high variability in the %VE results from the fact that cosine fits were made to only 20 data points (50 ms bins) for each period. **e** Box-and-whiskers plot showing change in %VE from the first 10 stimulus repeats to the last ten stimulus repeats in AAV-ReaChR-mCitrine treated retinae and wild-type controls. In degenerate treated retinae, %VE was significantly lower for the last ten stimulus repeats (*p* < 0.005), while in wild-type controls %VE was stable (*p* = 1). Please note in treated retinae that a decrease in %VE was observed in each of the observed neurons, though to a different extent. Data from non-degenerate wild-type mice are given in Suppl. Figure 5 together with an extended version of graph E

To analyze what would be the functional implications of this unphysiological response transience from the use of AAV-ReaChR-mCitrine as an optogenetic therapeutic for vision restoration we probed how neural responses to quasi-native visual stimuli could be encoded over extended periods of time. Retinae were exposed to a continuous, full-field cosine intensity modulated stimulus over 500 s (8 min 18 s, Fig. 4a). Most, but not all, cells appeared able to follow the stimulus over the entire protocol duration (Fig. 4b). Yet, in neurons #101 and #105, for example, the stimulus response is sharply demarked only over the first ~ 50 cosine-wave repeats but gets less defined and blurs from repeat ~ 180 onwards. To further quantify how the sharpness of the responses changes over time, the percentage of variance in the response that can be explained by the underlying stimulus function was calculated. Figure 4c and d show representative data obtained for one neuron exhibiting particularly stable responses and for one neuron exhibiting particularly fading responses. In these individual neurons, this observation of decreasing response sharpness was variably pronounced. On average, the percentage of variance explained decreased from a median of 35.05 [inter-quartile-range (IQR): 19.01–51.19]% during the first ten stimuli to 20.29 (IQR: 10.39–26.46)% during the last ten stimuli. Notably, a decrease was observed in each single neuron (Fig. 4e, Suppl. Figure 5d, *p* < 0.005, *n* = 15). For comparison, in wild-type retinae, there was no statistically significant change in the percentage of variance explained between the first and the last stimuli [first ten stimuli: 40.47 (IQR: 32.97–50.75)%, last 10 stimuli: 46.41 (IQR: 29.76–51.77)%, *p* = 1, regarding “ON”-type response neurons only; Fig. 4e and Suppl. Figure 5).

**Fig. 5:**
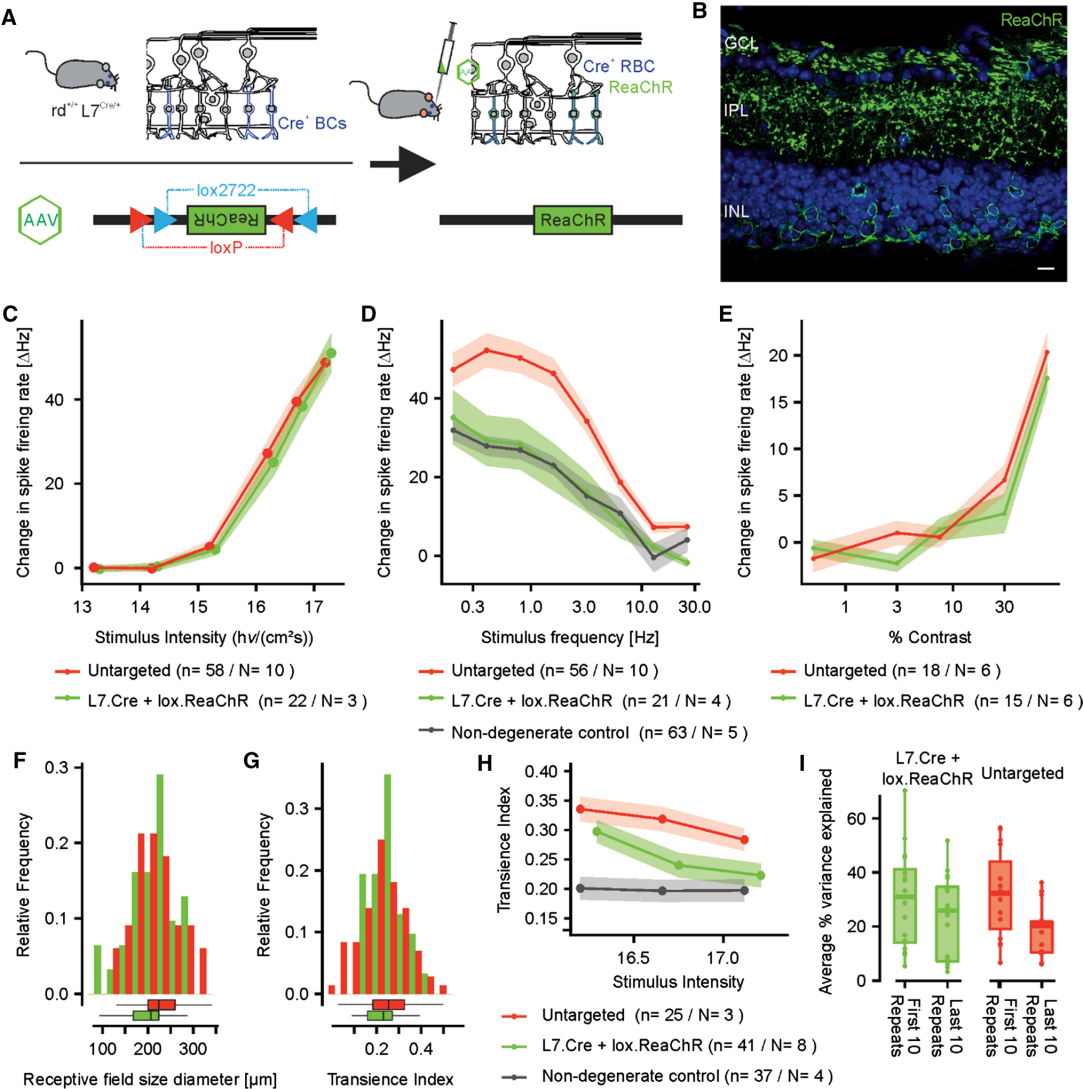
Comparison of light responses obtained in retinae of rd1 mice treated using ‘untargeted’ (ganglion cell favoring, red) or ‘rod-bipolar cell (BC) targeted’ (green) gene delivery approaches. Data underlying the graphs for untargeted delivery and wild-type retinae are the same as shown (Figs. 1, 2, 3, 4). A illustrates the experimental strategy used to achieve rod-BC-targeted delivery: *rd1*.L7Cre^±^ mice expressing cre recombinase in rod-BCs were injected with viruses carrying double-floxed inverse orientation (DIO) ReaChR (AAV-DIO-ReaChR-mCitrine). **b** Representative confocal cross-sectional scan obtained from a retina from an AAV-DIO-ReaChR-mCitrine treated *rd1*.L7Cre^±^ mouse, immunostained with anti-YFP (green). Please note, that ReaChR expression was also observed in a small portion of RGCs, which is in accordance with previous reports on L7 and L7Cre lines [26, 27]. (see also: Suppl. Figure 6) *GCL* Ganglion cell layer, *IPL* Inner plexiform layer, *INL* Inner nuclear layer. Blue: *DAPI* Scale bar 10 µm. Raw data available as Digital Supplementary Material. **c** Intensity-response curves are essentially indistinct between targeted and untargeted delivery (*p* = 0.11). **d** Changes in spike firing rate in response to flicker stimuli of increasing frequency. Responses obtained by rod-BC targeted delivery were similar to the responses observed in wild-type mice. **e** Changes in spike firing rate in response to a stepwise contrast modulation on a 9.8 × 10^16^ photons × cm^−2^ s^−1^ 568 nm background. Rod-BC-targeted delivery evoked significant changes in spike-firing rate only in response to a 75% contrast step (one sample Wilcoxon test). **f** Receptive field histograms: Receptive fields were significantly smaller in rod-BC targeted delivery as compared to untargeted delivery (*p* = 0.025). (**g**) Histogram of transience indices: While no difference of the means was observed between rod-BC targeted and untargeted delivery (*T* test: *p* = 0.16), values obtained in the rod-BC targeted delivery group showed signifyingly less spread (*F* test: 0.04). **h** transience indices for light stimuli of increasing intensity: In the untargeted and rod-BC-targeted delivery group, responses became increasingly transient with increasing light intensity. This effect was more pronounced in the rod-BC-targeted group. (*r* = -0.23, *p* = 0.009 vs. *r* = −0.21, *p* = 0.071). (I) Box-and-whiskers plot showing change in percentage of the variance explained (%VE) in the light responses to a continuous sine-wave stimulus from the first 10 stimulus repeats to the last 10 stimulus repeats for rod-BC targeted and untargeted delivery. %VE was significantly lower for the last 10 stimulus repeats as compared to the first in both treatment groups (*T* test: *p* < 0.05, each), but were slightly (though not significantly less pronounced for rod-BC-targeted delivery (*p* = 0.18)

To explore if the above established toolset could be sensitive enough to detect differences between distinct optogenetic gene-therapeutic strategies, we treated a cohort of *rd1*.*Opn4*^−/−^.*L7*^cre/+^ mice with AAV carrying floxed ReaChR [AAV-DIO-ReaChR-mCitrine, age at injection: 6 weeks, median age at tissue collection: 21.85 (21.10–24.93) weeks] [26, 27]. As expected, this resulted in a ReaChR expression pattern dominated by cells that had their soma at the outer portion of the inner nuclear layer (putatively rod-bipolar cells (rod-BCs), 15.12 [12.06– 20.56] % of cells in the inner nuclear layer, Fig. 5 A, B and Suppl. Figure 6). Besides rod-BCs also a small number of RGCs was transduced [7.00 (5.00–9.13)% of cells in the ganglion cell layer]. Hence, in accordance with previous reports [26], injection of *rd1*.*Opn4*^−/−^.*L7*^cre/+^ mice with AAV-DIO-ReaChR-mCitrine resulted in a rod-BC dominated, but not rod-BC exclusive transduction (Suppl. Figure 1 and Suppl. Figure 6). Retinae were then subjected to the same recording protocols as used above and compared to the retinae transduced with non-floxed AAV-ReaChR-mCitrine. After rod-BC-targeted delivery, light responsiveness could be observed in 39% of electrodes during MEA recordings (*p* = 0.74, Chi square for comparison with untargeted delivery). Light responses observed for rod-BC delivery showed a slight, but statistically insignificant delay in onset kinetics when compared to untargeted delivery, conceivably reflecting the additional synaptic processing [0.10 (0.05–0.20)s, *n* = 31, *p* = 0.76]. Peak response amplitudes [130.00 (90.00–170)/s, *n* = 31, *p* = 0.11] and half maximal irradiance response intensities (EC50: 1.87 [0.43—2.02] 10^16^ photons·cm^−2^ s^−1^, *p* = 0.11, Fig. 5c) were statistically indistinct. Frequency–response curves for rod-BC-targeted and untargeted delivery showed marked differences: curves obtained from AAV-DIO-ReaChR-mCitrine treated retinae revealed a flatter curve with lesser changes in spike firing rate for high frequencies. Observed responses were significantly different from zero up to 12.8 Hz (One-sample Wilcoxon test). Interestingly, the obtained curve was highly similar to that obtained when in recording from wild-type retinae (Fig. 5d). Moreover, receptive field diameters were significantly smaller for rod-BC-targeted as compared to untargeted delivery [205.95 (167.72–224.24) µm, *n* = 31, *p* = 0.025, Fig. 5f]. Response transience observed after rod-BC-targeted delivery was similar in mean but showed significantly less spread [0.23 (0.16–0.27], *p* = 0.16 (Wilcoxon test for difference in means) and *p* = 0.04 (Bartlett test of homogeneity of variances), Fig. 5g]. While these observations carefully point towards a functional advantage of targeting bipolar cells for vision restoration, responsiveness to “low” contrast stimuli was poorer for rod-BC-targeted delivery: A 75% contrast was required to evoke a statistically significant change in spike firing rate (One-sample Wilcoxon test, Fig. 5e). We additionally observed that cellular responses also in rod-BC-targeted retinae became more transient with increasing stimulus intensity, as we observed it for untargeted delivery (*r* = − 0.23, *p* = 0.01, Fisher’s z: *p* = 0.9, Fig. 5h). Finally, there was a non-significant trend of neural responses recorded form AAV-flox-ReaChR-mCitrine retinae to better maintain response fidelity over long-term stimulation with quasi-realistic stimuli: As for untargeted delivery, there was a decrease in %VE over the stimulus duration [first 10 stimuli: 32.24 (IQR: 19.01–44.09)%, last 10 stimuli: 30.96 (IQR: 13.96–41.27)%, *p* < 0.05]. Yet, the neuron-wise difference in %VE between first and last stimuli was markedly lower for rod-BC-targeted as compared to untargeted delivery [6.70 (2.913–11.03)%, *n* = 15 vs. 8.92 (4.25–18.86), *n* = 16]. However, this difference did not reach statistical significance (*p* = 0.18, Fig. 5i).

## Discussion

The present data provide a unique and quantitative functional assessment of the capabilities of optogenetic gene therapy to restore pattern forming visual responses at the output level of the retina in blind mice. We used AAV-ReaChR-mCitrine as a model optogenetic tool and combined multi-electrode array electrophysiology recordings with delivery of complex light stimuli. In this way, we show that in the degenerate retina of *rd1* mice accurate time and space-resolved neural responses can be restored. Data presented herein, highlight the capacity of optogenetic gene therapy but equally reveal several shortcomings of AAV-ReaChR-mCitrine-based approaches, namely a poor contrast encoding and a decay in response accuracy over prolonged stimulus episodes. We propose that the experimental toolset established in this work can be used to efficiently identify the optimal optogenetic tool, target cell population and time point for therapeutic intervention. It will moreover facilitate comparison of optogenetic gene therapy and alternative regenerative or prosthetic approaches for vision restoration.

### Functional capacity of optogenetic vision restoration

In this study, we were able to map receptive fields in the retinae of AAV-ReaChR-mCitrine treated mice that were comparable in size to those that we (and others) could map in untreated wild-type animals [28]. With a median diameter of 222 and 205 μm (for RGC and rod-BCs, respectively, ~ 0.05 mm^2^), these were slightly smaller than those observed in retinal ganglion cells of transgenic blind mice expressing channelrhodopsin-2 under the control of cre-recombinase [29] and those observed using chemical photoswitches [30]. Most notably, they were by approximately one order of magnitude smaller than those that could be obtained when using high-density sub- or epiretinal photovoltaic implants in mice [31]. This highlights the capacity of optogenetic approaches for vision restoration when compared to prosthetic retinal implants.

A second important observation made in this study was the relatively fast decay in neural response fidelity in treated neurons, evidenced by a significant reduction in the percentage of the variance in spike-firing-rate that can be explained by the underlying stimulus. We attributed this to the transient spike firing responses as recorded in most RGCs. Response transience was previously observed for microbial opsins in the context of optogenetic gene therapy. However, previous studies had interpreted the observed transience as consequence of the intrinsic membrane properties of the transduced RGCs (i.e., “transient” vs. “sustained” RGCs) [16, 6, 8]. The present data, rather suggest that transgene or transgene-delivery-related factors underlie response transience: transience indices (as a quantitative measure of response transience) were normally distributed, which would not be expected if membrane properties of discrete classes of RGCs were underlying the observed effect. Consistently, a non-normal distribution of transience indices was observed in recordings from non-degenerate wild-type retinae. Transience indices negatively correlated with stimulus intensity. In other words, the higher the stimulus intensity, the more transient the recorded responses. As expected, this was not the case in native wild-type retinae. Thus, one explanation for the observed transience could be the biophysical properties of the optogenetic tool itself—namely, channel inactivation/desensitization. This appears unlikely as ReaChR inactivation would be expected only for light-intensities higher than those used in this study [23]. Also, a lower spread in the observed transience would also be expected. A more likely explanation is that transgene-delivery-related factors like variable and high expression levels of the optogenetic tool cause response transience [32, 33]. In this, activation of an excess of channel-opsins might cause a rapid ion imbalance and thereby impede sustained firing by inducing a depolarization block-like effect.

In functional terms, the consequence of both response transience and loss in response fidelity over time which we observe, are that a ReaChR-based optogenetic vision restoration therapy may only enable treated patients to encode visual input for a restricted amount of time. After that, presumably, periods of darkness would be required for recovery. Notably, from the present data, it remains unclear if light responsiveness actually recovers to initial levels, or if long-term stimulation of AAV-ReaChR-mCitrine treated cells is associated with cell damage. Moreover, response fidelity was not systematically tested for different light intensities. This question will need further investigation including ex vivo electrophysiological, histopathologic as well as behavioral experiments. In any case, the present data highlight that optimization of the optogenetic tool, and/or delivery techniques are required. With regard to the optimal optogenetic construct, native mammalian opsins are attractive, as they use an intracellular signaling cascade expectably enabling more fine-tuned neural activation [e.g., 20,17,15]. Potential disadvantages of mammalian opsins, like the slower kinetics in particular in the case of melanopsin, for instance, may become an acceptable trade off, if they would enable long-term response fidelity. The experimental toolset presented in the present work will support comparison and optimization of different candidate optogenetic constructs.

### The value of sequestering native intraretinal signaling pathways

In the present work, we also explore the usefulness of the novel toolset by questioning if there was a measurable benefit of targeting retinal bipolar cells in an optogenetic vision restoration therapy. In theory, targeting these upstream second order neurons would retain a portion of the native retinal circuity potentially enabling the natural intraretinal signal processing to occur. Indeed, using the novel toolbox we found that when targeting rod-BCs several beneficial effects could be observed: flicker frequency–response curves were flatter as compared to untargeted, RGC-dominated delivery, very closely resembling our observations from wild-type non-degenerate retinae. Moreover, receptive fields were smaller, which would be consistent with a smaller dendritic field and speculatively preservation of some center-surround inhibition mechanisms. Also, the loss in response fidelity over sustained stimulus episodes was reduced; the reasons for this can only be speculated on. Yet, it is conceivable that bipolar cells with their primarily graded signal encoding are more capable of compensating the excessive ion-flux through ReaChR than the spike generating RGCs—which show a far greater propensity to enter states of depolarization block. All these observations indicate a functional advantage of targeting bipolar cells for vision restoration. Yet, it is also important to highlight that contrast sensitivity, which was relatively poor in recordings from retinae subject to untargeted delivery was worse after targeted delivery. Conceivably, the steep stimulus–response relationship of ReaChR in conjunction with the graded signal encoding in rod-BCs puts their ability to encode differences in light stimulus intensity within very narrow constraints. This illustrates how the selection of the optimal target cell population directly functionally interlinks with the selection of the optogenetic tool.

Interestingly, in non-degenerate retinae, rod-BCs signal via AII-amacrine and ON or OFF bipolar cells to RGCs, evoking sign-conserving and sign-inverting responses on ganglion cell level [34, 35]. Yet, after rod-BC-dominated delivery, we almost exclusively observe sign-conserving RGC responses. This is in keeping with the observations made in several other studies [14, 36, 8]. Yet, Mace et al. [12] did also manage to restore sign-inverting responses. It remains unclear if this was due to differences in the gene delivery approach or if potential genetic differences in the mouse line utilized play role. None the less, restoring also the sign-inverting circuity seems attractive to make maximum use of the intraretinal signal processing.

Indeed, wherever we compare between our ‘untargeted’ and the rod-BC-targeted delivery, it needs to be kept in mind that the ‘untargeted’ delivery approach resulted in a predominant, but not exclusive expression of ReaChR in RGCs. As it can be observed in (Fig. 1c), a very limited number of neurons in the INL was also transduced. A potential minor non-RGC contamination will therefore be included in the spike firing responses we recorded. In the opposite direction, this thought also applies to our rod-BC targeted delivery approach—though too much lesser extent: transgene expression can also be observed in a small number of ganglion cells (Fig. 5b) [26, 27].

### Implications for clinical trials

Clinical trials aiming to restore vision utilizing optogenetic approaches are currently emerging. To date, two studies (combined Phase I/IIa) have commenced using microbial opsins to restore vision in patients with end-stage retinitis pigmentosa (NCT02556736, NCT03326336 at clinicaltrials.gov.). The data acquired here originate from the output level of the retina and do not include any information from behavioral or cognitive level. However, while clinical trials are ongoing, the data presented herein are of particular interest for at least two reasons: (1) they suggest that at the retinal level relatively high-grade pattern vision, at least with regard to the two key aspects, temporal and spatial encoding, can generally be achieved. This indicates that optogenetic vision restoration has the capacity to provide benefit also to patients with partially preserved image forming vision, including individuals with macular dystrophies or widespread atrophic age-related macular degeneration [37]. (2) The data presented here equally highlight relevant functional limitations: poor contrast encoding and a significant loss of response fidelity upon sustained stimulation. Where stimulus enhancing goggles are employed, these limitations should be taken into consideration to optimize the signal delivered to the retina (e.g., goggles should not constantly transmit light but leave “recovery breaks” after shot intervals of time). These limitations may also not be ignored when designing or selecting functional endpoints for clinical trials to avoid that treatment success is overseen, e.g., if test paradigms employ insufficient contrast or are too long lasting. In this regard, high brightness fundus controlled perimetry devices are promising [38].

As much as the present data may help to select and design functional endpoints, they highlight the need to further explore potential safety-relevant aspects of optogenetic vision-restoration. For instance, in the case of ReaChR, a particular advantage is that it is able to function at light levels that are below the safety threshold [16]. However, the decay in light response fidelity over stimulation episodes as short as 8 min we observed conceivably represents a transgene mediated pathophysiological light effects as discussed above. Such considerations need to be taken into account before planning future clinical trials: candidate optogenetic tools could be studied utilizing protocols similar to those described herein in combination with detailed histopathologic analyses of retina of model animals that also address morphologic changes in transduced neurons.[c.f., 32] With regard to the running clinical trials, assessment of structural markers, e.g., monitoring by spectral-domain optical coherence tomography also from a safety perspective could informative.

### Capacity and limitations of stimulus protocols applied herein

Side by-side comparisons of candidate opsins are scarce and either only provide comparison of very similar opsins or only assess very basic functional parameters [9, 14, 18, 20]. The methodological strategy utilized here, employing MEAs to record neural responses from the output level of explanted retinae together with a system that allows for delivery of complex patterned light stimuli can serve to compare and benchmark the spectrum of candidate optogenetic constructs. This strategy is particularly attractive compared to behavioral readouts as it avoids interference with several confounders (e.g., fear responses to the very bright light when aiming to stimulate microbial opsins or unintended changes in overall irradiance when using LED array background illuminated mechanical optokinetic drums) that are often encountered in behavioral level tests. As it has been repeatedly shown that optogenetic gene therapy is capable of restoring cortical and behavioral level light responsiveness, (e.g., 17,15) assessment of visual responses on organ level now provides an efficient—and expectably more sensitive—way of comparing individual vision-restoring approaches. Remarkably, this ex vivo strategy could also be used to compare optogenetic gene therapy to alternative approaches including stem cell therapy or retinal prosthetics. It will allow identification of the optimal target cell population for an optogenetic vision restoration therapy and the best time point in the process of retinal degeneration for therapeutic intervention. In this context, it is interesting to see that the present data have been obtained from 8-month old mice, an age at which extensive remodeling of the retinal circuity has occurred [39]. In particular the well-defined receptive fields indicate that advanced remodeling does not critically impede vision restoration by optogenetic gene therapy.

Several vision restoring approaches (including optogenetic and prosthetic strategies) are sign inverting, i.e., response polarity at the retinal output level is inverse to the native situation, while others are not. Comparison of sign-inverting to sign-maintaining vision restoration strategies is generally possible with the experimental approach presented herein by only adjusting the analysis pipeline. Notably, while we know from retinal prosthetic implants that also sign-inverting approaches can generate optical sensations informative to the treated individual, it is currently unknown what the behavioral cost of this sign inversion is. Hence, it is likely that when comparing sign-inverting to sign-maintaining approaches only major differences can be translated into a likely perceivable difference for pattern forming vision.

Stimulus protocols used in this study were adjusted for the requirements of AAV-ReaChR-mCitrine induced photoresponses. With regard to receptive field mapping, they were therefore brighter than required to obtain optimal rod/cone responses in non-degenerate retinae. Even low levels of stray light may have stimulated adjacent cone photoreceptors in the wild-type mice and thereby increased the mapped receptive field. As we did not dark adapt the retinae between individual stimuli, it is also likely that we predominantly/exclusively mapped cone receptive fields. Data from wild-type mice presented in this manuscript therefore only serve for orientation and should not be misinterpreted as reflecting the full functional capacity of a non-degenerate retina. Moreover, stimulus protocols were designed to probe certain hallmark features of the neural signal at the output level of the retina. Yet, they are not capable of interrogating to what extent the functional diversity of retinal ganglion cells is maintained in treated retinae. While this does not affect the interpretation of the data obtained in the present work, more complex stimulus protocols, e.g., as recently employed by Baden et al*.* [28] may enable assessment of additional functional features of retinal ganglion cells elicited by optogenetic gene delivery in degenerate retinae.

In the present study, we have chosen a stimulus intensity 1 log-unit above the EC50 of ReaChR. One important reason for doing so was that we wanted to avoid stimulating at the steep side of the intensity-response curve to reduce variability of responses from cells with low levels of expression, and produce more consistent trial to trial responses. Notably, fine-tuning of the stimulus for the particular opsin under investigation will be required to support precise quantification of the maximal functional capacity of an individual optogenetic tool. Likewise, other parameters like stimulus intervals may require adjustment. Importantly, by analysing spike responses patterns to individual stimulus protocols the particular factors requiring fine-tuning can readily be identified.

To-date, most approaches to measure optogenetic vision restoration at the behavioral level effectively measure irradiance detection instead of complex, spatio-temporal encoded vision. Only few groups have managed to design devices that allow for gradual assessment of space-resolved pattern vision restored by—in particular microbial opsin based—gene therapy [18, 13,8, 21, 20]. Commonly, those devices are yet awaiting validation by independent groups. Utilizing novel behavioral test paradigms a recent study showed that optogenetic therapy using a cone-opsin allowed complex behavioral responses to patterned light stimuli to be restored [20]. However, utilization of behavioral essays in optogenetically treated mice usually requires laborious adjustment of various test parameters for the needs of the particular optogenetic tool [21]. Moreover, they only assess certain aspects of visual function (e.g., only spatial resolution or contrast) and putatively lack the resolution required to accurately analyse the various aspects of image-forming vision and compare responses between different optogenetic tools. While many factors will certainly influence the ultimate success of visual restoration approaches (e.g., central brain remodeling) it is clear that the ability of the retina to respond to distinct types of physiological light stimuli is the critical factor determining the ultimate capacity of these approaches to restore different aspects of functional vision (sensitivity, duration, spatial and temporal kinetics of responses for example). In other words, behavioral responses to complex light stimuli can be worse, but not better than what is encoded at the retinal output level. We suggest that the toolset we have developed—recording at the retinal output level—offers a powerful and efficient approach to compare the degree of visual restoration offered by various therapeutic approaches in a first instance. Successfully identified candidate optogenetic tools should then be moved forward towards assessment in specifically adjusted mouse behavioral essays and expression analyses in human retinal organoids [40]. Together, this will provide high-resolution functional, immediately relevant behavioral and cellular-level translatability data. Such a workflow may then efficiently deliver the evidence necessary to port the candidate optogenetic tool forward into a Phase I/II clinical trial.

## Conclusions

The present study describes a toolset that allows for efficient comparison of therapeutic approaches aiming to restore vision at the retinal output level. By employing this toolset, important quantitative information on the capacity and limitations of ReaChR to restore spatiotemporally encoded vision is provided. The protocols used herein can serve to prescreen, analyze and benchmark a spectrum optogenetic constructs for vision restoration. They provide an efficient approach to identify an optimal optogenetic tool and fine-tune further aspects of optogenetic gene therapy for vision restoration that could then be ported towards behavioral level analysis and finally into clinical trials.

## Materials and methods

### Animals

All experiments were approved by the University of Oxford Animal Welfare and Ethical Review Board and all procedures were conducted in accordance with the UK Home Office Animals (Scientific Procedures) Act 1986 (Project License 30/3371, Investigator License I0AEE55E7) and the ARVO Statement for the Use of Animals in Ophthalmic and Vision Research. Mice were housed under 12-h light / dark cycle at 21 °C, diet and water were available ad libitum. Mice used throughout this study were offspring of a colony maintained on a *Pde6b*^*rd1/rd1*^*. L7cre*^±^ background. *Pde6b*^rd1/rd1^ (*rd1*) mice show virtually complete rod degeneration at the age of 4–8 weeks, with ongoing cone degeneration (the latter comprising for only 3% of wild-type mouse photoreceptors) [24, 41]. *L7cre*^±^ mice express cre-recombinase under control of a rod bipolar cell-specific promoter [42, 26]. Only mice wild type or heterozygous for *L7cre* were used. Heterozygous mice are phenotypically normal. For some experiments mice additionally had the *Opn4* gene (coding for melanopsin, the photopigment of the inner retina) knocked-out. *Pde6b*^rd1/rd1^.*Opn4*^−/−^ mice lack all intrinsic retinal photoresponses and were used to rule out any contribution of residual native light responses.

### Virus production and intravitreal injection

Viruses carrying ReaChR were produced using methods described previously [43, 44]. Hek 293 T were transfected with pAAV2/2 (quad Y-F) (containing rep and cap genes), pAdDF6 (containing helper genes from adenovirus genome) and either AAV-ReaChR-mCitrine (containing red-shifted Channelrhodopsin with a C-terminally fused mCitrine under the hSyn promotor) or AAV-DIO-ReaChR-mCitrine (where the coding sequence was inverted and flanked by two lox sites), harvesting was performed 72 h later. Cell pellets were lysed and incubated with benzonase (50 U/ml, Sigma-Aldrich, St. Louis, MO, USA) to remove cell DNA contamination. AAV particles were then purified by iodixanol gradient ultracentrifugation and concentrated using Amicon Ultra-15 centrifuge filters (Merck-Millipore, Burlington, MA, USA). The titre of DNase1-treated virus was determined by standard-curve qPCR using primers designed to amplify a portion of the inverted terminal repeat sequence (ITR) as previously described (Primers: forward: 5′-GGA ACC CCT AGT GAT GGA GTT; reverse: 5′-CGG CCT CAG TGA GCG A) [45]. AAV-ReaChR-mCitrine and AAV-DIO-ReaChR-mCitrine were kind gifts from Roger Tsien (Addgene plasmid # 50,954; https://n2t.net/addgene:50954; RRID: Addgene_50954 and Addgene plasmid # 50,955; https://n2t.net/addgene:50955; RRID: Addgene_50955) [23].

For intraocular injection mice were anesthetized using isoflurane. Pupils were pharmacologically dilated and a 6 mm coverslip positioned on gel lubricant (Viscotears, Novartis, Basel, Switzerland) was applied to the cornea. 1 µl of virus at 2 × 10^11^ viral genomes/ml was injected into the vitreous using a Nanofil syringe with a 35-gauge needle (NANOFIL + NF35BV-2, World Precision Instruments Inc, Sarasota, USA) using a surgical microscope (M620 F20, Leica, Wetzlar, Germany) [17]. Only mice heterozygous for *L7cre* were used when injecting AAV-DIO-ReaChR-mCitrine.

### Multi-electrode array recordings

Multi-electrode array (MEA) recordings were performed as we described previously [17]. In brief, following enucleation, retina were dissected under dim red light (> 610 nm) and transferred to glass-bottomed MEA chambers (Multi Channel Systems, Reutlingen, Germany), with ganglion cell side facing down. MEA chambers (containing 60 electrodes, each 30 µm in diameter and spaced 200 µm apart) were placed into the MEA recording device (MEA1060-Inv; Multi Channel Systems) and positioned on the stage of an inverted Olympus IX71 microscope so that recording electrodes were located in the microscope light path. Retinae were perfused with Ames’ media bubbled with 95% O2/5% CO2 (pH 7.3) and maintained at 34 °C. Responses were recorded from an area of 4 × 4 electrodes that were within the light path of the pattern stimulator (see below). Recorded signals were collected, amplified, and digitized at 10 kHz using MC Rack software (Multi Channel Systems). Retinas were perfused for at least 30 min in darkness before beginning of the recordings.

### Generation of light stimuli

Light stimuli were generated by a Dual OptoLED light source (Cairn Research, Faversham, UK) equipped with 470 nm and 565 nm narrowband LEDs. Lights paths of both LEDs were fused into a single light path after passing motorized filter wheels containing neutral density filters (ThorLabs, Newton, USA) allowing for accurate, independent brightness control over five orders of magnitude. The common light path was then fed through a digital mirror device (“pattern stimulator”, Polygon400, Mightex Systems, Toronto, Canada) mounted to the fluorescence port of the Olympus IX71 and thereby enabling spatially precise high contrast delivery of complex light patterns. All devices were automatically controlled and synchronized by a Digidata 1440A (Axon Instruments, Molecular Devices, San Jose, USA) digital I/O board and a PC running WinWCP (J Dempster, Strathclyde University, Glasgow, UK) [46]. LEDs and Polygon400 were connected in parallel to the digital in ports of the MEA1060-Inv to assure accurate timing. PolyLite software (Mightex) was run in slave mode to control the Polygon400. Stimulus protocols were generated using Matlab R2016A and R2017A. Power of light stimuli (in microwatts per square centimetre) were measured at the sample focal plane using an in-line power meter (PM160T; ThorLabs). Power meter readings (in watts) were adjusted for the field of view of the pattern light stimulator (0.61 mm^2^), and units converted to photons × cm^−2^ s^−1^ using the Irradiance Toolbox [47]. Hence, a power meter reading of 0.42 mW corresponds to a light intensity of 68.85 mW × cm^−2^ s and a 565 nm photon flux of 1.96 × 10^17^ photons × cm^−2^ s^−1^.

### Stimulus paradigms

Six different light stimulus paradigms were used in this study and are summarized in (Table 1). Throughout the entire study, stimulus paradigms were run at 565 nm wavelength for all retina degenerate (treated and untreated) and at 470 nm for wild-type mice. A full brightness light pulse equals 1.96 × 10^17^ photons x cm^−2^ s^−1^ for 565 nm and 2.72 × 10^17^ photons × cm-^2^ s^−1^ for 488 nm. A recovery period of ~ 30 s was allowed between individual stimulus protocols. A longer recovery period of 20 min was allowed for the experiments in untreated *Rd1* mice (Suppl. Figure 2a) to capture native melanopsin responses.

**Table 1:** Summary of stimulus protocols and specific analysis strategies as employed in this study

No	Title	Stimulus protocol	Data processing
1	Full field stimulus	A 5 s period of darkness (= LED off) is followed by a 5 s full field 1.96 × 10^17^ photons x cm^−2^ s^−1^ stimulus and again a 5 s period of darkness	Peri-stimulus histogram of spike firing rate, calculated for bins of 100 ms. Where indicated, transience indices were calculated as the area under the curve, during the stimulus interval after standardization. A transience index of 1 thereby indicates a perfectly sustained response (i.e., maximum firing rate sustained over entire stimulus period)
2	Intensity response curve	400 ms full field stimuli of increasing intensity (1.96 × 10^13^ photons x cm^−2^ s^−1^—1.96 × 10^17^ photons × cm^−2^ s^−1^) with an inter stimulus interval (ISI) of 15 s	For each intensity: Spike firing rate = [*n* (spikes during stimulus) – *n* (spikes during interval of same duration before stimulus)]/(2* stimulus duration). Stimulus intensities for half-maximal responses were obtained by Hill-fits of recordings for the individual neurons
3	Flicker frequency response	Alternating episodes of 1.96 × 10^17^ photons × cm^−2^ s^−1^ stimuli and darkness of equal duration. Flicker frequency decreased stepwise in a square-root fashion from 0.25 Hz to 25 Hz. The total time stimuli of any frequency were delivered was constant at 5 s., i.e., a single 0.25 Hz stimulus was shown, but two 0.5 Hz stimuli, thereby leaving the total time of stimuli and darkness constant over all tested frequencies	For each stimulus frequency: [*n* (spikes during stimuli) – *n* (spikes between stimuli)]/5 s
4	Contrast modulation response	A stimulus of 50% of the maximum brightness (9.8 × 10^16^ photons x cm^−2^ s^−1^) was applied for 15 s. Brightness was then modulated around this level resulting in (positive) contrast steps of 0.5%, 3%, 7.5%, 30%, and 75% for a duration of 1 s each	For each contrast level: Spike firing rate = [*n* (spikes during current stimulus)/stimulus duration] – [*n* (spikes during 2nd half of previous stimulus)/(stimulus duration * 0.5)]. The rational for omitting the first half of the previous contrast step in the equation was to exclude any potential ‘tail’ activity arising from the previous contrast step. Only ON-type responses were analyzed
5	Receptive fields:	A sparse binary noise, applied as a 16 × 16-chessboard pattern (256 consecutive frames). Frame duration was 400 ms. Light intensity at bright fields was 1.96 × 10^17^ photons × cm^−2^ s^−1^ and contrast between bright a dark field was 1:1000. Projection area on focal plane (10 × objective) was 0.61 mm^2^, resulting in a pixel area of 2.38 × 10^–3^ mm^2^	Spike firing rate (calculated as for #2) obtained for any stimulus frame were multiplied with a 2d binary matrix representing the stimulus pattern during that particular frame to assign a stimulus location to any observed response. Resulting matrices were then collapsed and normalized. Only ON-type responses were analyzed
6	Intensity response curve with sustained stimuli	5 s full field stimuli of increasing intensity (5–0 ND) with an ISI of 20 s	Same as #2. Transience indices were calculated as described for #1
7	Sine wave protocol	A continuous full field stimulus was applied over 500 s. Stimulus intensity was a sine wave function with a period duration of 1 s and an amplitude of 0 ND on a dark background	Spike firing rate was binned in 50 ms intervals, normalized and re-arranged as a function of phase and period. % of variance explained was calculated performing ANOVA tests after cosine-fitting the data obtained for each stimulus period. Only ON-type responses were included into the statistical analysis

### Data processing and statistical analysis

Proprietary MEA recording files (*.mcd) were converted into *.bin files using the MC Data Tool (Multi Channel Systems). Converted flies were then imported into Matlab where they were bandpass filtered (0.3–3 kHz) and spike detection was performed. Standard spike detection threshold was set to three standard deviations of the trace but could be adjusted if necessary. Supervised spike sorting was then performed using custom-made Matlab-based software. In brief, this software performed K-means clustering and Silhouette analysis based on spike shape-defining parameters to estimate the number of individual neurons recorded from on each MEA electrode. Results were presented together with a two principal components representation of the dataset and permitted manual adjustment of the clustering results where required (e.g., marking clusters that represent artefacts rather than true spikes and discard them from further analysis). Results were stored as binary matrix (columns representing time points, rows representing individual neurons or the LED/Polygon400 trigger traces). Subsequent analyses were performed individually for the distinct stimulus protocols, as described in (Table 1). Time windows for capturing the responses were set according the response polarity (ON, OFF, ON–OFF). For ON–OFF responses, the ON component was analyzed. Non-ON type responses were excluded from further analysis in three protocols as indicated in (Table 1) to avoid interference with the analysis procedures. Proportion of ON responses analyzed was 85.7% for non-floxed AAV treated, 93.5% for floxed AAV-treated and 71.2% for wild-type retinae. Protocols for response detection were set up with the primary intention to capture ON responses. Hence there might be a minor bias towards under-detection of non-ON responses equally affecting all cohorts. Statistical analyses were performed in the R software environment for statistical computing, utilizing the ggplot2 package, and the appropriate statistical tests (for comparisons of means: Wilcoxon test, unless other ways specified). Error bands in graphs represent standard errors of the mean, unless otherwise indicated [48, 49].

### Immunohistochemistry

For histological analysis, mice were culled by cervical dislocation and tissue was collected and processed according to in-house standard procedures [50]. Whole eyes were fixed in 4% methanol-free paraformaldehyde (Thermo Fisher, Waltham, MA, USA) in phosphate buffered saline (PBS) for 24 h, transferred to 30% (w/v) sucrose in PBS and stored at 4 ºC for > 48 h. For preparation of retinal cryosections eyes were then transferred into Optimal Cutting Temperature medium (VWR, Lutterworth, UK) and stored at − 80 ºC until further processing. 18 µm tissue sections were prepared using a Cryotome FSE (Thermo Scientific). For immunostaining of retinal flat mounts, eyes were dissected as described above for MEA recordings. Fluorescent immunolabelling was performed using standard techniques as previously described [50]. Briefly, retinal sections were permeabilized in PBS with 0.2% Triton-X and blocked in PBS with 10% normal donkey serum (Sigma Aldrich, St. Louis, USA). Sections were subsequently incubated in chicken polyclonal anti-GFP, which also recognizes eYFP (GFP-1020, AVES Labs, Tigard, OR, USA, 1: 500) and rabbit polyclonal anti-cone arrestin (AB15282, Merck, Darmstadt, Germany) for 16 h at 4 °C diluted in 2.5% donkey serum in PBS. Secondary antibodies were donkey anti-chicken Alexa-488 (Jackson ImmunoResearch, West Grove, PA, USA) and donkey anti-rabbit Alexa-568 (Thermo Fisher) diluted 1: 2000 in PBS with 2.5% donkey serum and 1% Triton-X for 16 h at 4 °C. Retinal sections were viewed on a confocal microscope (LSM 710; Carl Zeiss AG, Oberkochem, Germany). Fluorescence of DAPI, Alexa 488 and Alexa 568 was sequentially recorded and superimposed by inbuilt software. Image processing (global linear adjustment of brightness and contrast) was performed using FIJI/ImageJ [51]. Unprocessed images from the main manuscript are also provided in full resolution as supplemental digital material [ 52].

## Electronic supplementary material

Below is the link to the electronic supplementary material.

Supplementary file1 (PDF 5520 kb)

## Data Availability

Data presented in this work will be made available upon request.
