# Supplementary material for: The functional characteristics of optogenetic gene therapy for vision restoration
Source: Cell Mol Life Sci. 2020 Jul 29;78(4):1597–613. doi: 10.1007/s00018-020-03597-6 (PMC7904736; doi:10.1007/s00018-020-03597-6)
Supplement: Supplementary file 1 — Supplementary file1 (PDF 5520 kb) [file 18_2020_3597_MOESM1_ESM.pdf]

Supplementary Materials:

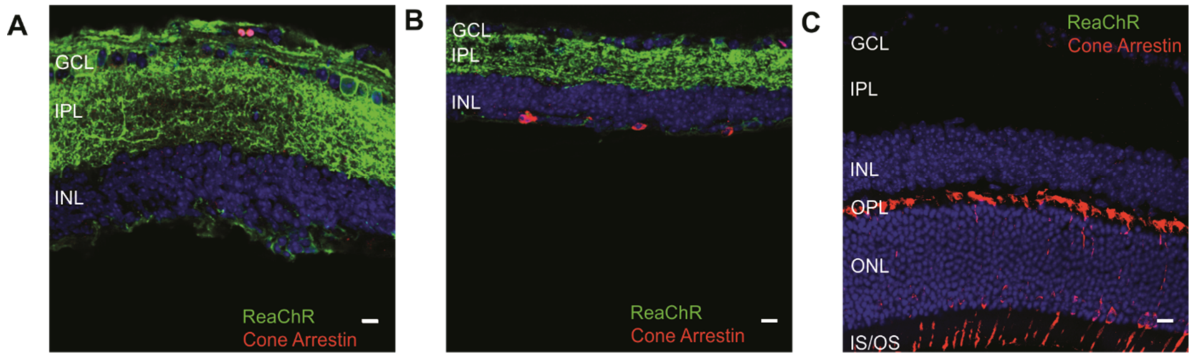

Suppl. Figure 1 (to Figure 1 D): Transduction pattern of AAV-ReaChR-mCitrine and cone survival in treated *rd1* retinae. A, B) Two confocal cross sectional scans obtained from an AAV-ReaChR-mCitrine treated retina of an *rd1* mouse, immunostained for AAV-ReaChR-mCitrine (anti-YFP, green) and cone photoreceptors (anti cone-arrestin, red). Transduced cells were predominantly situated in the GCL, but very cells in the INL were also transduced. Based on their localization, these appear to be mainly amacrine cells [52] A) Virtually no cone-arrestin positive cells could be observed in representative cross sections. B) only very occasionally residual cone-arrestin positive cells could be observed (exemplified herein on a cross section from the peripheral retina). Cone arrestin positive cells always lacked inner and outer segments. C) Cross section from an untreated non-degenerate wild type mouse for comparison showing the typical pattern of cone-arrestin staining. GCL: Ganglion cell layer, IPL: Inner plexiform layer, INL: Inner nuclear layer, OPL: Outer plexiform layer, ONL: Outer nuclear layer, IS/OS: inner and outer segment layers. Blue: DAPI. Scale bar: 10 µm.

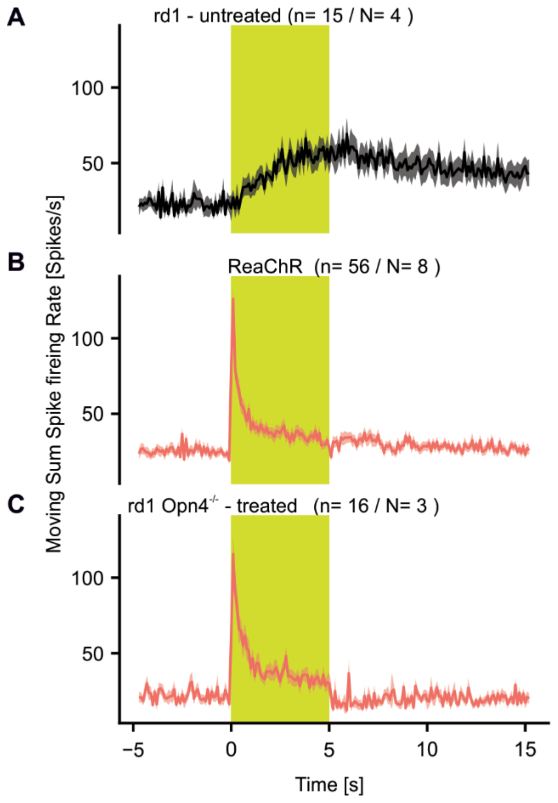

Suppl. Figure 2 (to Figure 1 A): Light responses to 1.96 x 10^{17} photons x cm-^{2}s^{-1}) 565nm light stimuli in treated and untreated *rd1* mice. A) In retinae from untreated *rd1* mice after a prolonged period of darkness slow and sustained light responses can be observed, representing native melanopsin activation. B) After intravitreal delivery of AAV-ReaChR-mCitrine, more rapid (Time to peak 5.80 [3.35 – 6.45]s vs. 0.10 [0.00 – 0.10]s, p<0.001), and more pronounced changes in spike firing rate (110.00 [85.00 – 120.00]/s vs. 135.00 [90.00 – 170.00]/s, p=0.02) are observed. C) In treated mice additionally lacking native melanopsin (*Pde6b*$^{rd1/rd1}$.*Opn4*$^{-/-}$) light responses were essentially indifferent from those in mice that expressed native melanopsin in terms of peak firing rate (105.94±9.11/s, p=0.87) and onset kinetics (0.10 [0.10 – 0.30]s, p=0.76).

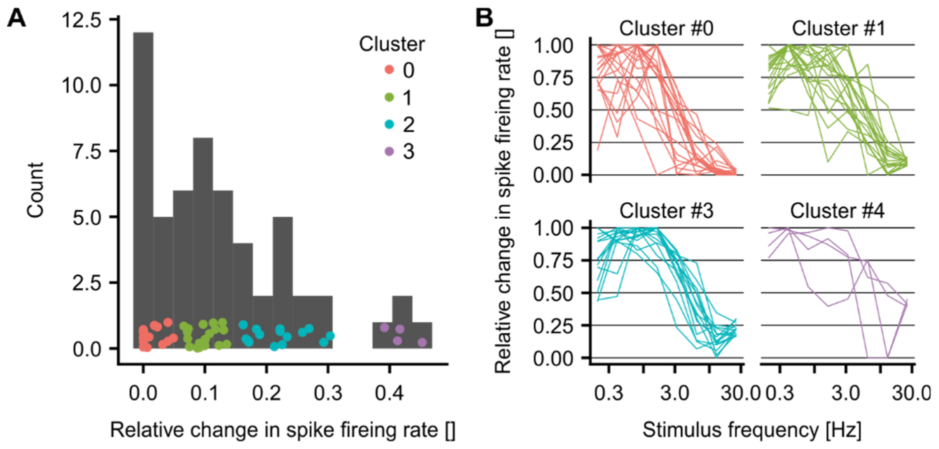

Suppl. Figure 3 (to Figure 2 A): Individual neuronal responses to increasing frequency flicker stimuli (1.96 x 10^{17} photons x $cm^{-2}s^{-1}$ 568 nm). A) Histogram (grey bars) and underlying single measurements (dots) of change in relative spike firing rate in individual neurons in response to a 25.4 Hz flicker stimulus as recorded from AAV-ReaChR-mCitrine treated retinae from *rd1* mice. Dot color indicates clusters as obtained by k-means clustering The number of clusters was chosen by the number of peaks observed in the histogram. Vertical positioning of dots is for visualization purposes only. While the majority of neurons show relative changes in spike firing rate of <0.1, two clusters of neurons exhibit more pronounced changes of >0.15 and >0.35, respectively. B) Traces for each individual neuron over the entire stimulus range, grouped by assigned clusters as in A. As a trend, neurons assigned to lower order clusters (low relative change in spike firing rate in response to a 25.4 Hz flicker stimulus) had a left-shifted frequency-response relationship (i.e. encoded high stimulus frequencies less efficiently).

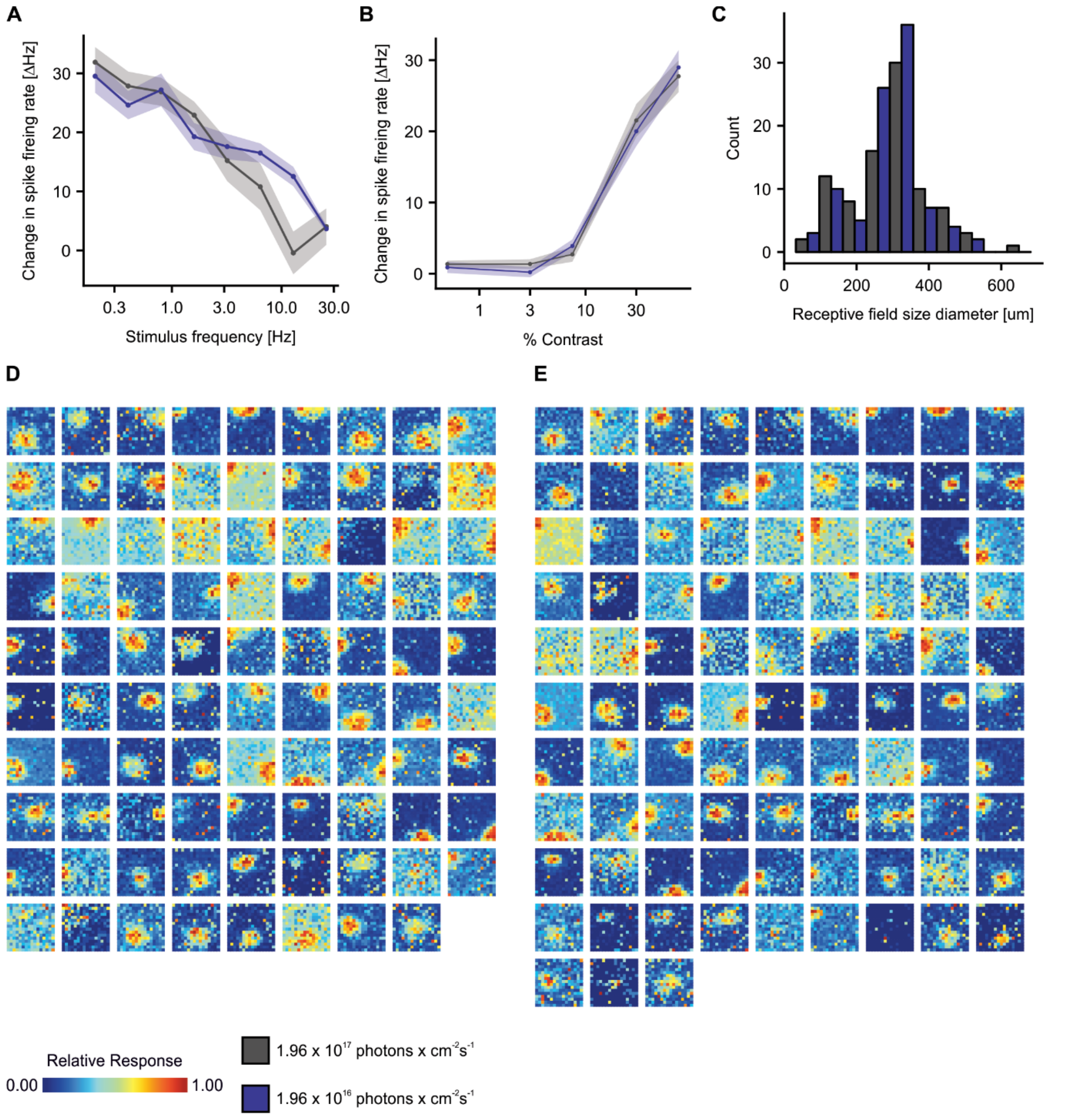

Suppl. Figure 4 (to Figure 2 E – G): Spatiotemporal characteristics recorded from wild type retinae using different stimulus intensities and protocols as presented in Figure 2. A) Change in spike firing rate in response to a 568 nm flicker stimulus of increasing frequency. B) Change in spike firing rate in response to a stepwise contrast modulation. C) Histogram of receptive field size diameters. D, E) Receptive field maps (resolution: 2.38 x 10-3 mm2/pixel), obtained in response to a 1.96 x 10^{17} photons x $cm^{-2}s^{-1}$ (D) and 1.96 x 10^{16} photons x $cm^{-2}s^{-1}$ (E). Responses recorded at 1.96 x 10^{17} photons x $cm^{-2}s^{-1}$ and on a 9.8 x 10^{16} photons x $cm^{-2}s^{-1}$

568 nm background, respectively, are presented for comparison and replicate those shown in **Figure 1**. For responses recorded at 1.96 x 10^{16} photons x $cm^{-2}s^{-1}$ n= 62 / N= 5 in A and n= 33 / N= 4 in B.

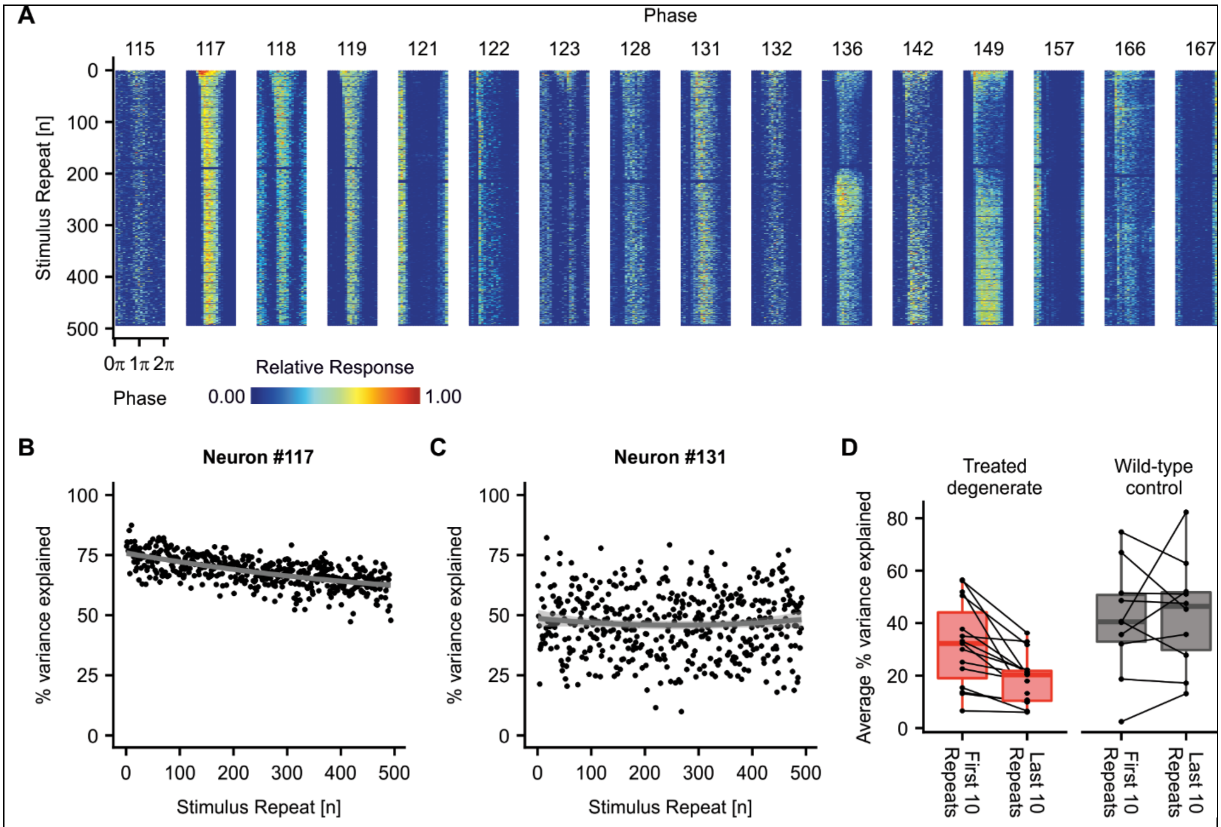

Suppl. Figure 5 (to Figure 4): Long-term responsiveness to quasi-realistic light stimuli in non-degenerate retinae from wild type mice. Data in this figure are for comparison to data from AAV-ReaChR-mCitrine treated retinae as presented in **Figure 4** B-D. A) Colour-coded response raster-plot for individual neurons (recorded from N=3 retinae) showing neuron-wise normalized responsiveness to a cosine-amplitude modulated stimulus (T=1, peak intensity: 1.96 x 10^{17} photons x $cm^{-2}s^{-1,}$ 568 nm) over 500 repeats (dark blue: minimum firing rate, red: maximum firing rate – see **Figure 4** A for details). On (e.g. neuron #117), Off (e.g. #121) and On-Off (e.g. #118)-type responses can be observed. Spike firing responses generally appear more stable over time, though occasionally transient alterations in response intensities were observed (#136, #149) B, C) Percentage of variance explained (%VE) over time, obtained

from ANOVA test to fits to cosine-functions to the responses for each stimulus repeat in three exemplary neurons. D) Box-and-whiskers plot showing change in %VE from the first 10 stimulus repeats to the last 10 stimulus repeats AAV-ReaChR-mCitrine treated retinae and wild type controls. Figure is identical to that shown in **Figure 4** E, but additionally showing the change in %VE for the individual neurons. In degenerate treated retinae %VE was significantly lower for the last 10 stimulus repeats ($p<0.005$), while in wild type controls %VE was stable ($p=0.691$). Note in treated retinae that a decrease in %VE was observed in each of the observed neurons, though to a different extent.

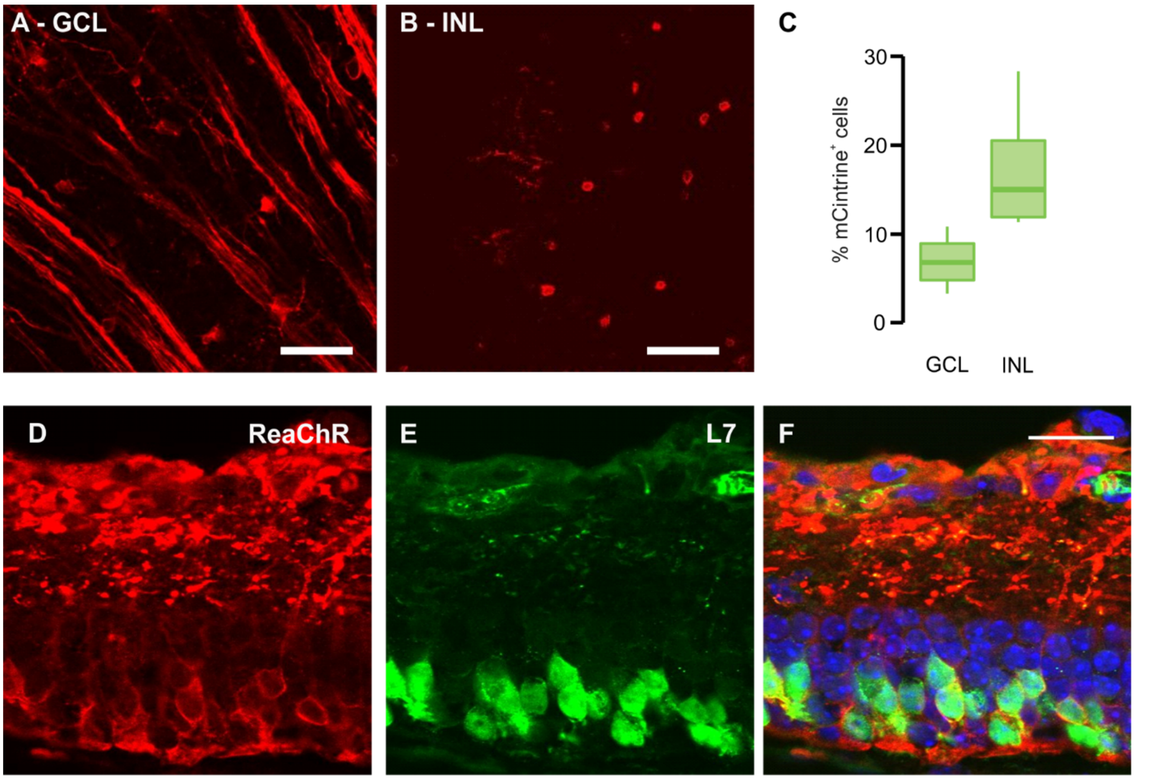

Suppl. Figure 6 (to Figure 5): Transduction pattern of AAV-DIO-ReaChR-mCitrine in *rd1.Opn4*$^{-/-}$*.L7*$^{cre/+}$ mice. A, B) Representative confocal sections acquired at the level of the ganglion cell layer (A, GCL) and the Inner nuclear layer (B, INL) from retinal flat-mounts obtained from AAV-DIO-ReaChR-mCitrine injected rd1.Opn4-/-.L7cre/+ mice and immunostained for ReaChR-mCitrine. C) Box-and-whiskers plot showing the percentage of cells transduced in the GCL and the INL, respectively. Data shown were obtained from four retinae of four mice. For each retina, the percentage of cells transduced in the GCL and the INL layer in was calculated for four independent areas. D-F: Cross section from a treated retina (unrelated to those shown in A-C and **Figure 5 B**) showing ReaChR-mCitrine (D,F) immunoreactivity in red and L7 (sc137064, Santa Cruz Biotechnology, USA; E,F) immunoreactivity in green. Scale bar: 50µm.
